# Three Doses of COVID-19 Vaccines: A Retrospective Study Evaluating the Safety and the Immune Response in Patients with Multiple Sclerosis

**DOI:** 10.3390/jcm12134236

**Published:** 2023-06-23

**Authors:** Giorgia Teresa Maniscalco, Daniele Di Giulio Cesare, Valerio Liguori, Valentino Manzo, Elio Prestipino, Simona Salvatore, Maria Elena Di Battista, Ornella Moreggia, Antonio Rosario Ziello, Vincenzo Andreone, Cristina Scavone, Annalisa Capuano

**Affiliations:** 1 Multiple Sclerosis Regional Center, “A. Cardarelli” Hospital, 80131 Naples, Italy; gtmaniscalco@libero.it (G.T.M.); d.cesaresm@gmail.com (D.D.G.C.); valentino.manzo@aocardarelli.it (V.M.); elio.prestipino@aocardarelli.it (E.P.); simona.salvatore@aocardarelli.it (S.S.); elena.dibattista@aocardarelli.it (M.E.D.B.); ornellamoreggia@gmail.com (O.M.); antonioziello@libero.it (A.R.Z.); 2 Neurological Clinic and Stroke Unit, “A. Cardarelli” Hospital, 80131 Naples, Italy; vincenzo.andreone@aocardarelli.it; 3 Department of Experimental Medicine, University of Campania “Luigi Vanvitelli”, 80138 Naples, Italy; valerio.liguori@unicampania.it (V.L.); annalisa.capuano@unicampania.it (A.C.); 4 Regional Center of Pharmacovigilance and Pharmacoepidemiology of Campania Region, 80138 Naples, Italy

**Keywords:** COVID-19, mRNA-based vaccine, safety, multiple sclerosis, AEFI, observational study, three doses

## Abstract

Since the beginning of the mass immunization of patients with multiple sclerosis (MS), many data on the efficacy and safety of COVID-19 vaccines have been produced. Considering that MS is an autoimmune disease and that some disease-modifying therapies (DMTs) could decrease the antibody response against COVID-19 vaccines, we carried out this retrospective study with the aim to evaluate the safety of these vaccines in terms of AEFI occurrence and the antibody response after MS patients had received the third dose. Two hundred and ten patients (64.8% female; mean age: 46 years) received the third dose of the mRNA-based COVID-19 vaccine and were included in the study. Third doses were administered from October 2021 to January 2022. The majority of patients (*n* = 193) were diagnosed with RRMS and EDSS values were ≤3.0 in 72.4% of them. DMTs most commonly used by included patients were interferon Beta 1-a, dimethyl fumarate, natalizumab and fingolimod. Overall, 160 patients (68.8% female) experienced 294 AEFIs, of which about 90% were classified as short-term, while 9.2% were classified as long-term. The most commonly reported following the booster dose were pain at the injection site, flu-like symptoms, headache, fever and fatigue. Regarding the immune response, consistently with literature data, we found that patients receiving ocrelizumab and fingolimod had lower IgG titer than patients receiving other DMTs.

## 1. Introduction

During the COVID-19 pandemic, recommendations from the Italian Ministry of Health highlighted the need to take care with much more attention of the frail population, including those suffering from chronic diseases, such as multiple sclerosis (MS), who were recognized as extremely vulnerable people and consequently being part of the highest-priority categories [1].

Thus, since the approval of COVID-19 vaccines and the beginning of the mass immunization of MS patients, many data on the efficacy and safety of these vaccines in a real-life context have been produced. In this context, our research group (composed of neurologists, pharmacologists, psychologists, data managers and statisticians) has been actively engaged in the monitoring of MS patients who received flu and COVID-19 vaccines, documenting the main features of patients receiving vaccines in the COVID-19 era and the efficacy and safety profile of these vaccines [2,3,4]. In particular, in November 2022 we published the results of the first retrospective study in which we analyzed the safety profile of the mRNA COVID-19 vaccines in patients with MS vaccinated with the Pfizer-BioNTech vaccine at the MS Center of the Hospital A.O.R.N. A. Cardarelli [5]. In this study, 310 MS patients received the first dose and 288 the second dose. Patients were mainly diagnosed with Relapsing-Remitting Multiple Sclerosis (RRSM) and the majority of them were receiving a disease-modifying therapy (DMT) during the study period, mainly interferon beta 1-a, dimethyl fumarate, natalizumab and fingolimod. More than nine hundred Adverse Events Following Immunization (AEFIs) were identified, of which 539 were after the first dose of the vaccine and 374 after the second dose. The majority of these AEFIs were classified as short-term and were mainly represented by pain at injection site, flu-like symptoms and headache. 

After the publication of this study, many of the patients included received the third dose of the vaccine, and for some of them the immunologic response was monitored in terms of Anti-SARS-CoV-2 IgG value. Considering that MS is an autoimmune disease whose pathology can be explained by an altered immune system [6], and that some DMTs, especially CD20 depleting agents such as ocrelizumab and sphingosine-1-phosphate receptor modulator (S1PRM) such as fingolimod, seem to be able to decrease the antibody response against COVID-19 vaccines [7], and given the collection of new data among our MS patients, we carried out this retrospective study with the aim to evaluate the safety in terms of AEFI occurrence and the antibody response after the third dose of COVID-19 vaccines in people with MS.

## 2. Methods

### 2.1. Study Design

This was a retrospective observational study carried out with the aim to assess the safety profile of mRNA-based COVID-19 vaccines and the antibody response among MS patients at the MS Centre of the Cardarelli Hospital (Naples). Third doses were administered from October 2021 to January 2022. 

### 2.2. Demographic and Clinical Data Collection

The following data were retrieved: demographic data (MS diagnosis, age, gender); mRNA-based COVID-19 vaccine (date of vaccination, type of vaccine, vaccine batch); medical and clinical history, DMTs, all AEFIs that occurred after vaccination, patients’ general clinical course, values of Anti-SARS-CoV-2 IgG (BAU/mL). The blood samples were collected during the routine clinical practice at least two weeks after the third vaccine dose.

We carried out a descriptive analysis of all identified AEFIs in terms of time occurrence and preferred term (PT). Regarding the time of occurrence, AEFIs were classified as short-term when they occurred within 72 h of the first, second and booster dose of the vaccine and as long-term when they occurred within 20 days of the first, second and booster dose of the vaccine.

### 2.3. Statistical Analysis

Categorial variables were described as frequency and percentage, while continuous variables were reported by their mean and standard deviation. Shapiro–Wilk test was used to assess the normality of data distribution. In the case of not normal distribution, no parametric test was applied.

For difference between DMTs in terms of SARS-humoral response, we used one-way ANOVA with Gamess-Howell (no parametric post hoc test) for differences between groups. *p* value less than 0.05 was considered significant.

### 2.4. Ethics

The retrospective study was reported to the Ethic Committee of A.O.R.N. A. Cardarelli/Santobono-Pausilipon. No written informed consent was necessary for the study conduction based on its retrospective nature.

## 3. Results

### 3.1. Overall Safety Results

Clinical and demographic characteristics of enrolled patients are reported in Table 1. We enrolled 210 MS patients (64.8% female; mean age: 46 years) who received the third dose (booster dose) of the mRNA-based COVID-19 vaccine. As for previous doses [5], all patients received the Pfizer-BioNTech vaccine. In addition, 91.9% of patients (*n* = 193) were diagnosed with RRMS, 5.7% of patients (*n* = 12) with Secondary Progressive Multiple Sclerosis (SPMS) and 1.9% (*n* = 4) with Primary Progressive Multiple Sclerosis (PPMS). The mean disease duration was 11.5 years (data not shown). EDSS values were ≤3.0 in 72.4% of patients, followed by equal 3.5–5.5 in 16.2% of patients, and ≥6.0 in 11.4% of patients. With regard to DMTs, many patients (*n* = 36; 17.1%) were receiving interferon Beta 1-a, followed by dimethyl fumarate (*n* = 34; 16.2%), natalizumab (*n* = 33; 15.7%) and fingolimod (*n* = 22; 10.5%) (Table 1).

**Figure 1:**
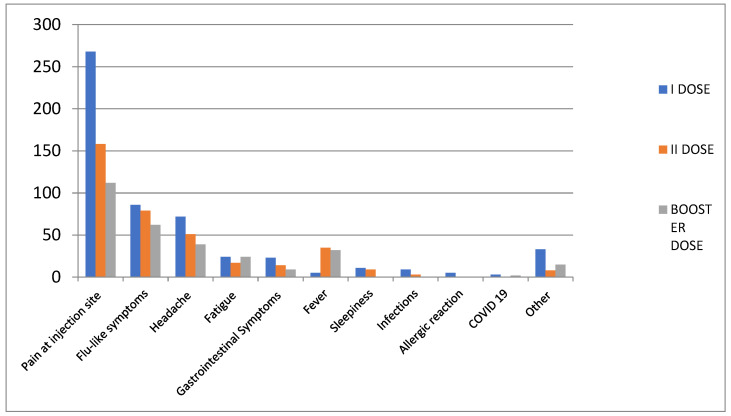
Distribution of Adverse Events Following Immunization (AEFIs) by Preferred Term (PT).

During the study period, 160 patients (68.8% female) experienced at least one AEFI (data not shown). In particular, 294 AEFIs occurred, of which about 90.8% were classified as short-term, while 9.2% were classified as long-term (Table 2). Looking at the type of AEFIs, the most commonly reported following the booster dose were pain at the injection site (*n* = 112; 38.0%), flu-like symptoms (*n* = 62; 21.0%), headache (*n* = 39; 13.3%), fever (*n* = 32; 10.9%) and fatigue (*n* = 24; 8.2%) (Figure 1). More in detail, the most common short-term AEFIs after booster dose were pain at the injection site (*n* = 111; 41.6%), flu-like symptoms (*n* = 56; 20.9%) and headache (*n* = 34; 12.7%). Fever (*n* = 32; 12%) was more frequent after the second dose of the vaccine and the booster dose than after the first vaccine dose. However, compared to the second booster dose, fatigue symptoms were similar following the administration of the booster dose and following the administration of the first dose of the vaccine (Table 3). 

### 3.2. Immune Response Results

After having received the third dose of the vaccine, 60 patients (28.6% of the cohort, 55% females) underwent laboratory tests, including the detection of SARS-CoV-2 IgG level (Table 1). The IgG test was performed after a mean of 33.1 days (±9.3), ranging from 15 to 60 days. The mean of Anti-SARS-CoV-2 IgG level following the booster dose was 8152 (±4750).

**Figure 2:**
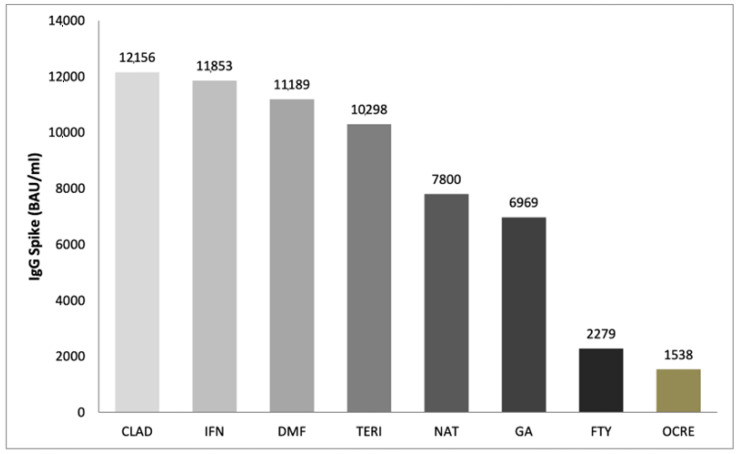
IgG Spike (BAU/mL) post booster dose in different DMTs. CLAD: Cladribine; IFN: Interferon B 1-a; DMF: Dimethyl Fumarate; TERI: Teriflunomide; NAT: Natalizumab; GA: Glatiramer Acetate; FTY: Fingolimod; OCRE: Ocrelizumab.

A difference in IgG level by DMTs was found. In particular, significant differences were found between: interferon Beta 1-a vs. ocrelizumab (10,315; *p* < 0.001) and fingolimod (9573; *p* < 0.001). Natalizumab showed higher levels of Anti-SARS-CoV IgG than ocrelizumab (6262; *p* = 0.019) and fingolimod (5521; *p* = 0.045). Lastly, patients treated with dimethyl fumarate had higher IgG titer of Anti-SARS-CoV-2 than ocrelizumab (9651; *p* < 0.001) and fingolimod (8910; *p* < 0.001) (Figure 2). 

Among patients for whom IgG level were available, 42 (70%) experienced short-term AEFIs. Of these patients, 25 were female (59.5% of total) and 17 were male (40.5%); mean EDSS was higher in patients who reported short-term AEFIs (2 vs. 1.1), with lower mean age (39 vs. 42).

## 4. Discussion

Since the beginning of the COVID-19 vaccination campaign among MS patients, a huge amount of clinical and laboratory data has been collected, representing a valuable source of information for a frail population such as MS patients. Since then, many research groups started to collect and analyze these data, providing new evidence on the efficacy and safety of COVID-19 vaccines among MS patients. For instance, a group of Italian researchers [8] compared the COVID-19 course and outcomes in MS patients on ocrelizumab and fingolimod after receiving the third dose of mRNA vaccine vs. patients on natalizumab. They enrolled 290 patients, of whom 79 were treated with natalizumab, 126 with ocrelizumab and 85 with fingolimod. Their results showed that patients who had COVID-19 on ocrelizumab and fingolimod were more symptomatic with higher hospitalization rates compared to patients on natalizumab. Overall, the results supported the effectiveness of the third booster dose of mRNA-Vax against severe forms of COVID-19 in patients treated with ocrelizumab and fingolimod. 

We carried out a retrospective study to examine the safety profile and the antibody response in 210 MS patients who received the third dose of a mRNA COVID-19 vaccine. More in detail, we carried out a descriptive analysis of the AEFIs by time of occurrence and PT.

Overall, our analysis showed that the mean age of 210 patients was 46.3 years, and almost 60% were females. These results are consistent with those of previous studies; indeed, as reported also by Harbo HF et al., women are more likely than men to get MS (the prevalence ratio of MS in women to men is 2.3–3.5:1) [9].

In our cohort, a total of 160 patients (76.2%) had at least one AEFI. Of these patients, almost 70% were female. In line with this finding, Peter Yamoah et al. reported that COVID-19 vaccine-induced AEFIs were more frequently observed in women than men and in patients of the age group 18–64 years [10]. Other studies reported similar difference in AEFIs distribution by gender [11,12].

RRMS was the most prevalent MS subtype among patients included in our study with interferon Beta 1-a, dimethyl fumarate and natalizumab the DMTs most frequently prescribed. These results are in line with previous findings [2,3,4,5] but also with another study that investigated the antibody response after the third dose of COVID-19 vaccine in MS patients affected by various MS subtypes [13]. According to our opinion, the distribution of drug use among MS patients may be related to the diverse population included in our study, especially in terms of age and MS type.

In our study, the most commonly reported AEFIs following the booster dose were pain at the injection site, flu-like symptoms, headache, fever and fatigue. Comparing our descriptive analysis of AEFIs with previous results [5], flu-like symptoms and fever were more frequent after the second dose of the vaccine, and in this analysis, following the booster dose than after the first dose. In most cases, these AEFIs are not serious and resolve on their own. Events occur mostly on the vaccination day or the day after. In line with our results, Sapir Dreyer-Alster et al. conducted a study on 211 MS patients (62% female, 74.8% treated with different DMTs) who received the third dose of the BNT162b2 vaccine. Following vaccination, no anaphylactic or life-threatening reactions occurred. The most commonly reported AEFIs were muscle or joint discomfort, fatigue, localized pain at the injection site and fever. Headaches and dizziness occurred in 7.6% and 1.9% of patients, respectively [14]. Regarding the occurrence of headache, Castaldo M et al. carried out a systematic review and meta-analysis of 84 articles (accounting for 1.57 million participants) to assess the pooled incidence of post-vaccine headache (after first and second dose) by vaccine type. In line with our results, headache was the third most common AE and a two-fold risk of developing headache within 7 days from injection was found. As reported by study’s authors, patients developing a new-onset headache should be carefully monitored for the risk of developing cerebral venous thrombosis, especially those with risk factors, such as thrombocytopenia, anti-platelet factor 4 antibodies, and multiple organ thrombosis [15]. Even though the mechanism underlying the occurrence of post-vaccination headache is currently unknown, this symptom could be a consequence of a homoeostasis disorder deriving from humoral and cellular immunity induced by the vaccine, which are characterized by an increase in inflammatory cytokines (i.e., interferon gamma, interleukin-6, CXC ligand 10) leading to a plethora of symptoms that also include headache [16,17].

We aimed also to investigate the antibody response among 60 MS patients who underwent—during routine clinical practice—laboratory tests, including SARS-CoV-2 IgG. According to this analysis, we found a difference in IgG level for different DMTs (ANOVA test, *p* < 0.001). Significant differences were found for the comparison interferon Beta 1-a vs. ocrelizumab (10,315; *p* < 0.001) and interferon Beta 1-a vs. fingolimod (9573; *p* < 0.001). As reported by Pitzalis M et al. [18], who evaluated the SARS-CoV-2 response after the second dose of the COVID-19 vaccine among 912 Sardinian MS patients and 63 healthy controls, humoral response to BNT162b2 was significantly influenced by these specific DMTs. In addition, Garjani et al. showed that fingolimod and ocrelizumab are to blame for the reduced immune response following COVID-19 vaccinations [19]. Similarly, findings were reported by Baba et al. [13]. Wu X et al. [7] carried out a meta-analysis of 48 studies published until March 2022 to evaluate the risk of impaired response to vaccination among 6860 patients with MS receiving DMTs. An attenuated serologic response was observed in patients treated with anti-CD20 (OR = 0.02, 95% CI: 0.01–0.03) and S1PRM (OR = 0.03, 95% CI: 0.01–0.06) after full vaccination compared with patients not receiving any DMT. No other significant associations between other DMTs and humoral response to SARS-CoV-2 vaccines were found by the authors’ study. Another study [20] found out that the effects of fingolimod on cellular immunity persisted for more than 2 years after a change to ocrelizumab; thus, clinicians should consider the possible failure to provide protection against SARS-CoV-2 when switching from fingolimod to ocrelizumab. König et al. evaluated the safety and immunogenicity profiles of the third dose of mRNA COVID-19 vaccine among MS patients already receiving fingolimod or anti-CD20 therapy. The results highlighted a better antibody response among patients who received anti-CD20 therapy than those who received fingolimod and that a higher absolute lymphocyte count was linked to both a better antibody response and more side effects [21]. In the same way, Achiron et al. compared the efficacy profile of the third dose of the COVID-19 vaccine respect to MS non-responder-patients treated with fingolimod. Specifically, a total of 20 patients were randomized into two groups, 10 patients in the fingolimod-continuation and 10 patients in the fingolimod-discontinuation. One month after receiving the third dose positive SARS-CoV-IgG antibodies were detected in two patients in the fingolimod-continuation group vs. eight patients in the fingolimod-discontinuation group, suggesting that fingolimod discontinuation is associated with beneficial humoral immune protection [22]. Lastly, Madelon et al. carried out a study where 20 patients that received ocrelizumab had a robust T-cell response recognizing spike proteins from the Delta and Omicron variants after vaccination. Following the third dose of the vaccine, there was an increase in the number of both CD4 and CD8 T cells that responded, showing that it was possible to improve individuals who had an undetectable memory response after the initial vaccination series [23].

## 5. Strengths and Limitations

The main limitations of our study include its retrospective and monocentric nature and the small sample size. In addition, the assessment of IgG response was used as a measure of assumed humoral immunity even though we are aware that antibody levels are not fully predictive of protection against infection and that the protective immune response to SARS-CoV-2 could also depend on T-cell responses. As we previously reported [5], considering that we cannot exclude the lack of important clinical data, our findings should be considered exploratory and interpreted with caution.

Nevertheless, we have provided new evidence collecting and analyzing clinical and laboratory data on the safety of the third dose COVID-19 vaccines in a frail population such as MS patients. As for previous studies, data collection and analysis have been performed by a multidisciplinary team of neurologists, pharmacologists, statisticians, and data managers with a long-standing experience in the management of studies focusing on the monitoring of the safety profile of medicines and vaccines [24,25,26]. 

## 6. Conclusions

In conclusion, our results indicated that the booster dose of the Pfizer-BioNTech vaccine was safe for MS patients, being associated with AEFIs already detected in the general population and with previous vaccine doses and shown in our previous publications. Specifically, in this study the booster dose was associated with 294 AEFIs which occurred in 160 patients (mainly women) and the most common AEFIs (mainly classified as short-term) were injection-site reactions, flu-like symptoms, headache, fever and fatigue. After having received the third dose of the vaccine, 60 patients underwent laboratory tests, including the detection of SARS-CoV-2 IgG levels. Consistently with literature data, we found that patients receiving ocrelizumab and fingolimod had lower IgG titer than patients receiving other DMTs.

## Figures and Tables

**Table 1:** Clinical and demographic variables of patients with multiple sclerosis who received booster dose COVID-19 vaccination.

Study Cohort	Patients Who Received the Third Dose	Patients with Blood Test Collected
Total patients	210	60
Female, *n* (%)	136 (64.8)	27 (45)
Male, *n* (%)	74 (35.2)	33 (55)
Mean age (years) ± sd	46.3 ± 13.4	39.6 ± 10.5
MS type, *n* (%)		
RRMS	193 (91.9)	59 (98.3)
SPMS	12 (5.7)	1 (1.7)
PPMS	4 (1.9)	-
PRMS	1 (0.5)	-
Disability by EDSS, *n* (%)		
≤3.0	152 (72.4)	55 (91.7)
3.5–5.5	34 (16.2)	3 (5.0)
≥6.0	24 (11.4)	2 (3.3)
DMTs, *n* (%)		
ocrelizumab	20 (9.5)	8 (13.4)
fingolimod	22 (10.5)	7 (11.7)
interferon beta 1-a	36 (17.1)	9 (15)
natalizumab	33 (15.7)	11 (18.3)
dimethyl fumarate	34 (16.2)	11 (18.3)
cladribine	12 (5.7)	6 (10)
glatiramer acetate	15 (7.1)	3 (5)
teriflunomide	23 (11)	5 (8.3)
interferon beta 1-b	6 (2.9)	-
methotrexate	1 (0.5)	-
rituximab	3 (1.4)	-
alemtuzumab	1 (0.5)	-
Untreated	4 (1.9)	-

RRMS: Relapsing Remitting Multiple Sclerosis; SPMS: Secondary Progressive Multiple Sclerosis; PPMS: Primary Progressive Multiple Sclerosis; PRMS: Primary Relapsing Multiple Sclerosis; EDSS: Expanded Disability Status Scale; DMTS: Disease-Modifying Therapies.

**Table 2:** Distribution of Adverse Events Following Immunization (AEFIs) by time of occurrence (short- and long-term) and vaccine’s dose.

	Total	First Dose	Second Dose	Booster Dose
Study population	310	310	288	210
All AEFIs, *n*	1207	539	374	294
Short-term AEFIs, *n* (%)	1057 (87.6)	438 (81.3)	352 (94.1)	267 (90.8)
Long-term AEFIs, *n* (%)	150 (12.4)	101(18.7)	22 (5.9)	27 (9.2)

**Table 3:** Distribution of Adverse Events Following Immunization (AEFIs) by time of occurrence and Preferred term (PT) among MS patients.

	Short-Term AEFIs	Long-Term AEFIs
Any adverse events, *n* (%)	267	27
Pain at injection site	111 (41.6)	1 (3.7)
Flu-like symptoms	56 (20.9)	6 (22.2)
Headache	34 (12.7)	5 (18.5)
Fever	32 (12)	-
Fatigue	17 (6.4)	7 (25.9)
Gastrointestinal symptoms	8 (3)	1 (3.7)
Other symptoms	9 (3.4)	5 (18.5)
Infection with SARS-CoV-2 after vaccination	-	2 (7.5)

## Data Availability

The data presented in this study are available on request from the corresponding author. The data are not publicly available due to privacy and ethical reasons.
